# The Hospitals of the Women’s Homœopathic Association

**Published:** 1887-06

**Authors:** Ad. Lippe

**Affiliations:** Philadelphia


### THE HOSPITALS OF THE WOMEN'S HOMŒOPATHIC ASSOCIATION OF PENNSYLVANIA,

### TWENTIETH STREET AND SUSQUEHANNA AVENUE.

AD. LIPPE, M. D., PHILADELPHIA.

On page 177 of the May number of THE HOMŒOPATHIC PHYSICIAN will be found a complete explanation of the resignation of eight lady physicians upon the visiting staff of the Hospitals of the Women's Homœopathic Association of Pennsylvania. The Managers of this Association have been compelled to respond to charges and accusations preferred against them in the public press by the resigning eight lady physicians and their sympathizers. As both the County Society and the misnamed Hahnemann Medical College have openly approved of the action of the eight resigning lady physicians, it is high time that the *motives* of these bodies be scrutinized and the implied insults offered the noble Managers of the Association be resented. The County Society and the eight lady physicians on a strike have resorted to the press to lay their grievances before the public; the managers of the Women's Homœopathic Association have, therefore, been forced to answer through the press also. We hold to the good old French proverb: "*Mieux vaut laver le linge sale en famille.*"

If the aiders and abettors of these eight complaining lady physicians had heeded this proverb they would not have published their case so extensively and thus have damaged themselves by it. For an investigation was thus stirred up that shows how wrong-doing requires singular modes to make it appear right. Their own confessions are of sufficient weight to expose their proceedings to the disgust and indignation of the community.

For ourselves, in conformity with the advice of the foregoing French proverb, we prefer to address ourselves to the profession through the pages of THE HOMŒOPATHIC PHYSICIAN. These lady physicians at the Women's Homœopathic Hospital introduced medical treatment, not homœopathic, into the Hospital: crude drugs in massive doses; disinfectants rejected by the allopathic school, stimulants rejected by Homœopathy, and, finally, "Castor oil" for purgative purposes. The Lady Managers, whose duty it was and is to see to it that in a professedly homœopathic hospital only homœopathic treatment should be administered to the sick, first kindly remonstrated with the erring lady physicians, but were met by some of them with the assertion that they, the physicians, were the only responsible persons, and, *therefore*, the Managers, as non-medical persons, should not interfere with their treatment. The Managers of the Association and of the Hospital are persons fully competent to determine what *is* homœopathic treatment and what is *not* homœopathic treatment, and under their charter it is their bounden duty, it is a trust conferred upon them by the people who give them a charter as homœopathists, to see to it that none but homœopathic means should be employed in the hospitals under their care; and let it be said here, once for all, that the Managers of the Women's Homœopathic Association know what Homœopathy means; that by their own individual experience they became convinced that the methods of Hahnemann, if honestly followed, produced better healing results than any other means in vogue, including the methods of those clamoring for the perversion of Homœopathy into Eclecticism. These philanthropic ladies desired to bestow on the public in general in the Women's Hospital the blessings of a truly homœopathic treatment, which was not to be obtained in any other hospital in this city. When they finally accepted the resignation of the striking doctors they did their full duty. If there were no longer homœopathic physicians to be found (as the strikers conjectured), the Hospital under its present management was still not likely to be turned over to the eclectics. These eclectics found themselves sadly disappointed when the strikers were locked out and a staff of faithful homœopaths were found to be ready to take their places. These eight physicians, grieving over the acceptance of their resignations (strike), now laid their case before the County Society, and this Society appointed at once a Committee of Inquiry. Now comes the farcical part of the case. Upon this Committee was put one of the disappointed women doctors: a perfectly novel proceeding which will no doubt amuse our brethren of the old school, who will make capital out of such a departure from ordinary rules of proceeding. The Committee did not, could not, make any inquiry, for in less than five minutes after their appointment they had *reported*, finding the eight strikers fully justified in their course of action. Without even attempting to hear the other side, they sustained the complainants. Next morning the papers contained the proceedings of the County Society, with the four names of the Committee of Inquiry, and their findings.

People are accustomed to the spectacle of whitewashing reports made by packed committees in political circles; but even in such cases the Committee always holds out at least a pretense of making proper inquiry and hearing of both sides. In the case before us the Committee appointment was a perfect farce. What then was the motive of these resigning doctors? Their apparent motive was to introduce into the Hospital a mode of practice not in harmony with the teachings of Homœopathy, but in harmony with the teachings and practice of the Hahnemann Medical College and its dispensary and hospital. These lady physicians attempted to justify their departure from Hahnemann's methods by quoting from a lecture I delivered at the Hospital in which I did say: "The whole scale, from the crude natural substances up to the higher and highest infinitesimals, should be open to the choice and the practice of every sensible and candid person." Does this declaration even impliedly justify the application of unhomœopathic remedies in crude large doses? Does it justify the pernicious local treatment with crude drugs? Does it justify the least departure from Hahnemann's methods? What I did say was necessary, as the eclectics parade themselves before the people as low potency men, and designate such physicians as are following Hahnemann's methods as high potency men, and assert that the only difference existing between the eclectics and the Hahnemannians turns on the potency question. It is apparent that this is a false issue altogether. The error has been very much augmented by certain persons who argue that any substance, even a nosode, becomes very homœopathic if highly potentized, and that all other necessary conditions for the choice of a curative remedy may be safely set aside if the nosode is only *highly potentized.* A physician who follows Hahnemann's methods implicitly will surely learn to prefer highly potentized drugs, while another physician who more and more departs from the teachings of the master will be as unsuccessful with highly potentized remedies as with low potencies, and must surely become an out and out eclectic. The County Society meeting at the Hahnemann Medical College was the Society selected by the eight lady physicians who had failed to introduce eclecticism into the Hospital of the Women's Homœopathic Association. The very fact that the County Society met at the building of the Hahnemann College was evidence enough that the Society indorsed the Hahnemann College as a homœopathic college.

The Hahnemann Medical College is by its own showing to all intents and purposes an eclectic college. Under the original charter the trustees (none of whom are medical men and certainly not members of the Faculty) were enjoined to have taught in that College all collateral sciences appertaining to medicine but "*especially*" Homœopathy. When eclectics gained positions in this *then* truly and *especially* homœopathic college, under false professions, of course, they caused the charter to be changed, and had that one word *especially* stricken out, and in its stead was inserted "*also.*" From that time forward we had the spectacle of the Hahnemann Medical College under eclectic teachings. The majority of the trustees of this now burlesque College are to this day the Faculty of the College and their tools —all medical men.

Appended to this burlesque College the leading eclectics propose a hospital to be given them under false pretenses, *a homœopathic hospital under eclectic treatment*—if the past is to be taken as a criterion for the future. *The Women's Hospital Association,* believing, no doubt, to do a charitable work, are sadly deceived by these men moving to have a homœopathic hospital attached to an eclectic college. The motive of opposition to the hospitals of the *Women's Homœopathic Association,* where it becomes evidently impossible to pervert Homœopathy into electicism, is now fully apparent.

Some very good persons have been deceived by the frequent advertisements of the Women's Hospital Association. Supposing that they were furthering the interests of Homœopathy and aiding the Women's Homœopathic Hospital, they have contributed to the support of the Association. But they were deceived by the close similarity of names, and they soon discovered that their contributions are going to the support of another association that is identified with eclecticism. This reminds us of an old eclectic trick years ago, when an eclectic college sought to use the reputation of a time-honored College, the University of Pennsylvania, by a close imitation under the name of the Pennsylvania University. It took ten years of litigation before this vile institution was wiped out. Its pupils survive! Behold their work in the above confounding of the names of two associations for hospital work.

But I hear the hissings of the galleries and perceive the smell of *eggs*, therefore, let the curtain fall on the *first* act.

We call attention to the publication of the first part of an exposition of the horrid teachings in a so-called homœopathic college (in Gotham?), by our old veteran, Dr. P. P. Wells, in the May number of THE HOMŒOPATHIC PHYSICIAN. We hope that Dr. Wells will exhibit more of the pernicious and wicked teaching at misnamed homœopathic colleges. We promise to follow suit, as we have a good deal to say yet about the abominable and treasonable teachings of a set of brazenfaced perverters of Hahnemann's Healing Art, by him called Homœopathy. There is a demand for homœopathic physicians and for homœopathic treatment, and the eclectics may remember that there is a universal law which governs demand and supply, and that a spurious or shoddy article furnished will invariably be rejected by those who know what the genuine article is and demand it. The Women's Homœopathic Association do furnish the genuine article, and no attempt to palm off a spurious article and condemn the action of a noble association *without* even listening to them will succeed, and, as we have shown that the motive of the County Society in sustaining the disgraceful conduct of these eight—now even nine—women was a mere blind to also sustain the *eclectic* Hahnemann Medical College, aided by the Women's Hospital Association, in their intention to palm off the spurious article, that article, spurious and adulterated, will surely meet the fate of all such articles while the genuine true Hahnemann Healing Art is offered to the community. The spurious article will be rejected by all well-informed persons; the spurious article may, for a time, deceive and be accepted by the ignorant, but it will not wear.

